# POCT Detection of *Pseudomonas aeruginosa* by PGM and Application of Preventing Nosocomial Infection of Bronchoscopy

**DOI:** 10.1155/2024/8062001

**Published:** 2024-09-05

**Authors:** Tao Wen, Houqi Ning, Yinping Yang, Jinze Zhang

**Affiliations:** ^1^ Department of Thoracic Surgery The Fourth Hospital of Hebei Medical University, Shijiazhuang 050011, China; ^2^ School of Food and Biotechnology Xihua University, Chengdu 611743, China; ^3^ Medical Department Beijing Jiahengyongtai Technology Co Ltd, Beijing 100036, China

## Abstract

**Background:**

The primary pathogen responsible for bronchoscope contamination is *Pseudomonas aeruginosa*. Conventional techniques for bronchoscopy disinfection and pathogen identification methods are characterized by time-consuming and operation complexly. The objective of this research is to establish a prompt and precise method for the identification of *Pseudomonas aeruginosa*, with the ultimate goal of mitigating the risk of nosocomial infections linked to this pathogen.

**Methods:**

The magnetic nanoparticles (MNPs) were synthesized in a single step, followed by the optimization of the coating process with antibodies and invertase to produce the bifunctionalized IMIc. Monoclonal antibodies were immobilized on microplates for the specific capture and enrichment of *Pseudomonas aeruginosa*. Upon the presence of *Pseudomonas aeruginosa*, the monoclonal antibodies, the test sample, and the IMIc formed sandwich structures. The subsequent addition of a sucrose solution allowed for the detection of glucose produced through invertase hydrolysis by a personal glucose meter, enabling quantitative assessment of *Pseudomonas aeruginosa* concentration.

**Results:**

TEM image demonstrates that the MNPs exhibit a consistent spherical shape. NTA determined that the grain diameter of magnetic nanoparticles was 200 nm. FTIR spectrum revealed the successful modification of two carboxyl groups on the MNPs. The optimization of the incubation pH of the microplate-coated antibody was 7. The optimization of the incubation time of the microplate-coated antibody was 2 h. The optimization of the ligation pH for the polyclonal antibody was 5. Reaction times of polyclonal antibodies linked to magnetic beads was 1 h. The pH of invertase linked by magnetic beads was 4.

**Conclusion:**

This article presents a novel qualitative and quantitative immunoassay for point-of-care monitoring of *P. aeruginosa* utilizing PGM as a readout. The PGM represents a convenient and accurate quantitative detection method suitable for potential clinical diagnostic applications.

## 1. Introduction

*Pseudomonas aeruginosa*, a Gram-negative pathogen, is frequently linked to nosocomial infections, infections in individuals with compromised immune systems, and persistent infections in patients diagnosed with structural lung disorders such as cystic fibrosis (CF). *Pseudomonas aeruginosa* is frequently implicated in nosocomial infections, presenting as pneumonia, surgical site infections, urinary tract infections, and bacteremia. The prevalence of *P. aeruginosa* among all healthcare-associated infections is estimated to be between 7.1% and 7.3% [1, 2]. Pneumonia is the primary site of *P. aeruginosa* infection and is the most prevalent Gram-negative pathogen identified in nosocomial pneumonia cases. Notably, the prevalence of *P. aeruginosa* infections has been steadily rising over the last ten years [3, 4]. *Pseudomonas aeruginosa* is responsible for a notably higher proportion of health medical-associated infections. A comprehensive observational point-prevalence study revealed that *P. aeruginosa* constituted 16.2% of infections among patients in intensive care units, with respiratory sources being the predominant site of *P. aeruginosa* infection [5]. Healthcare-associated pneumonia (HAP) and ventilator-associated pneumonia (VAP) impose a substantial burden on the healthcare system, contributing to as much as 22% of all healthcare-acquired infections [6].

Endoscopes play a crucial role as diagnostic and therapeutic tools in contemporary medicine. Nonetheless, inadequate disinfection practices applied to endoscopes can potentially lead to the emergence of healthcare-acquired infections (HCAIs). Numerous instances have been documented wherein various types of endoscopes have been implicated in the occurrence of such outbreaks. Bronchoscopy is a widely utilized diagnostic and therapeutic modality in the field, particularly within the domain of respiratory medicine. Bronchoscopy assumes a distinctive function in the identification and management of tracheobronchial lesions, pulmonary space-occupying pathologies, and various other ailments [7–10]. The utilization of electronic bronchoscopy is limited by its exorbitant cost and intricate design, necessitating multiple individuals to employ it repeatedly within a brief timeframe. Therefore, the cleaning and disinfection of the bronchoscope must be standardized to prevent cross-infection.

Bronchoscopes have been consistently identified as the most commonly implicated type of endoscope in harboring pathogens [11, 12]. Given the potentially life-threatening consequences associated with *Pseudomonas aeruginosa* infections in patients, it is imperative to rigorously implement regular and meticulous cleaning protocols for bronchoscopes. The conventional method for disinfection testing of bronchoscopes involves extracting a neutralizing sample solution from the biopsy orifice of the disinfected endoscope using a sterile syringe. This solution is then inoculated onto a sterile nutrient agar plate for culture and enumeration. However, this detection approach is characterized by a slow and time-consuming process. Consequently, there is an immediate requirement for the development of a rapid detection method for bronchoscopic pathogens. PGM has demonstrated a strong potential for application in rapid detection within various domains. However, previous research has predominantly focused on employing PGM for the detection of foodborne pathogenic microorganisms, primarily in the context of food safety. Conversely, the utilization of PGM for medical purposes, particularly within hospital settings, remains relatively limited, with a notable scarcity in the detection of common microbial pathogens. The objective of this study is to develop a detection approach for internal quality control of nosocomial infection in the bronchoscopy room by leveraging the rapid, sensitive, and user-friendly outcomes provided by a glucose sensor. This method aims to prevent the incidence of aeruginosa-related nosocomial infections.

## 2. Materials and Methods

### 2.1. Source of Materials

*Pseudomonas aeruginosa*, and other pathogenic bacteria, including *Escherichia coli*, *Klebsiella pneumoniae*, *Staphylococcus aureus*, and yeast, were acquired from an industrial microbial culture collection center in China. These microorganisms were obtained with their pathogenic gene deactivated, rendering them nonpathogenic. Monoclonal and polyclonal antibodies targeting *P. aeruginosa* were procured from Shanghai Huiyun Biotechnology Co., Ltd. 1-Ethyl-3-(3-dimethylaminopropyl)carbodiimide hydrochloride (EDC), N-hydroxysulfosuccinimide sodium salt (sulfo-NHS), 2-(N-morpholino)ethanesulfonic acid (MES), polyoxyethylene sorbitan monolaurate (Tween-20), sucrose, and trehalose were purchased from Sangon Biotech Co., Ltd. (Shanghai, China). Invertase (300 U/mg), bovine serum albumin (BSA), tris(2-carboxyethyl)phosphine hydrochloride, and 6-succinimidyl-4-(N-maleimidomethyl) cyclohexane-1-carboxylate were purchased from Sigma-Aldrich Co. (St. Louis, MO, USA). PGM and special strips (On Call Plus Ⓡ) were purchased from Acon Co., Ltd. (Zhejiang, China).

### 2.2. The Immobilization of the Antibody on the Microplate

As a first step, the polystyrene microplate was noncovalently coated with anti-*P. aeruginosa* antibodies. The microplate was incubated at 4°C in a humid chamber for 2 h with a coating solution of anti-*P*. *aeruginosa* antibodies (PBS, pH 7.0). The microplate was rinsed thoroughly and then incubated in 1% BSA for 1 hour to decrease nonspecific binding. Afterwards, the microplate was repeatedly washed, and *P. aeruginosa* was detected.

### 2.3. Preparation of Invertase-MNPs-IgG Conjugates (IMIcs)

To produce invertase-MNPs-IgG conjugates (IMIcs), a one-pot methodology was utilized to synthesize magnetic nanoparticles (MNPs) in accordance with the procedure outlined by Jia et al. [13]. Typically, a solution was prepared by dissolving 0.68 mmol of trisodium citrate, 1.2 g of sodium acetate, and 4.0 mmol of FeCl3·6H_2_O in 20 mL of ethylene glycol. After heating and stirring to obtain a yellow turbid solution, it was transferred into an autoclave lined with Teflon and heated at 200°C for 10 hours. *T* Subsequently, the precipitate was separated with a magnet and the magnetite nanoparticle was washed multiple times with ethanol and deionized water until the solution turned colorless and transparent, followed by drying at 80°C under a vacuum.

MNPs were conjugated with invertase and detection antibodies. Following the dispersion of 2 mg of MNP in 500 *µ*L of activation buffer (MEST, 10 mM, 0.05% Tween-20, pH 5.0), 2.5 mg and 2.5 mg of EDC and NHS were added, and the mixture was stirred for 30 min. Then, the mixture was transferred to 500 *µ*L of borate buffer (BB buffer, 20 mM, pH 5.0), and the products were washed twice and dispersed. MNP surface was first incubated with 20 *μ*g of anti-*P*. *aeruginosa* polyclonal antibody under stirring at 4°C for 1 h. Then, MNPs were incubated with 100 g of invertase for 1 hour at 4°C in MES buffer (10 mM, pH 4.0). We washed and redispersed the obtained IMIc into 100 *µ*L of storage solution (BBT, 5 mM, 0.05% Tween-20, 0.1% BSA, pH 7.2).

### 2.4. Establishment of Testing Procedures

**Figure 1:**
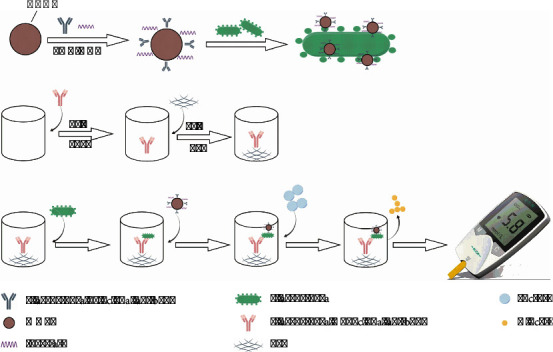
Flow chart.

Figure 1 depicts the schematic representation of the immuno-PGM sensing system employed for the detection of target *P. aeruginosa*. The system utilizes the anti- *P. aeruginosa* capture antibody-coated microplate. Initially, microtiter plates were loaded with 50 *μ*L of *P. aeruginosa* standard or sample in pH 7.0 phosphate buffer and incubated at 37°C for 30 minutes. Following the washing step, 50 *μ*L of the previously prepared IMIc suspension was added to each well and incubated for 30 minutes at 37°C. Subsequently, each well was injected with 50 *μ*L of sucrose (0.5 g/mL) in pH 7 phosphate buffer after the plate was washed again. Finally, PGM was used to detect glucose concentration.

### 2.5. Validation of Clinical Samples

We prepared a 10 mM glucose solution and measured it three times using a PGM. If the variation (CV) was ≤ 5%, PGM was said to have a good stability. The *Pseudomonas aeruginosa* culture solution, with a concentration of 10^7^ CFU/ml, was administered to the bronchoscope, followed by rinsing with 10 ml of normal saline. The presence of *Pseudomonas aeruginosa* was subsequently assessed using a glucose sensor POCT.

The Pseudomonas aeruginosa culture solution, with a concentration of 107 CFU/ml, was administered onto the bronchoscope. Subsequently, employing this novel disinfection procedure, the disinfection duration was halved and then further reduced by three-quarters. Following disinfection, the bronchoscope was rinsed with 10 ml of normal saline. The resulting rinsate was subjected to *Pseudomonas aeruginosa* content analysis using a glucose sensor point-of-care test (POCT).

## 3. Results

### 3.1. Characterization of MNPs

**Figure 2:**
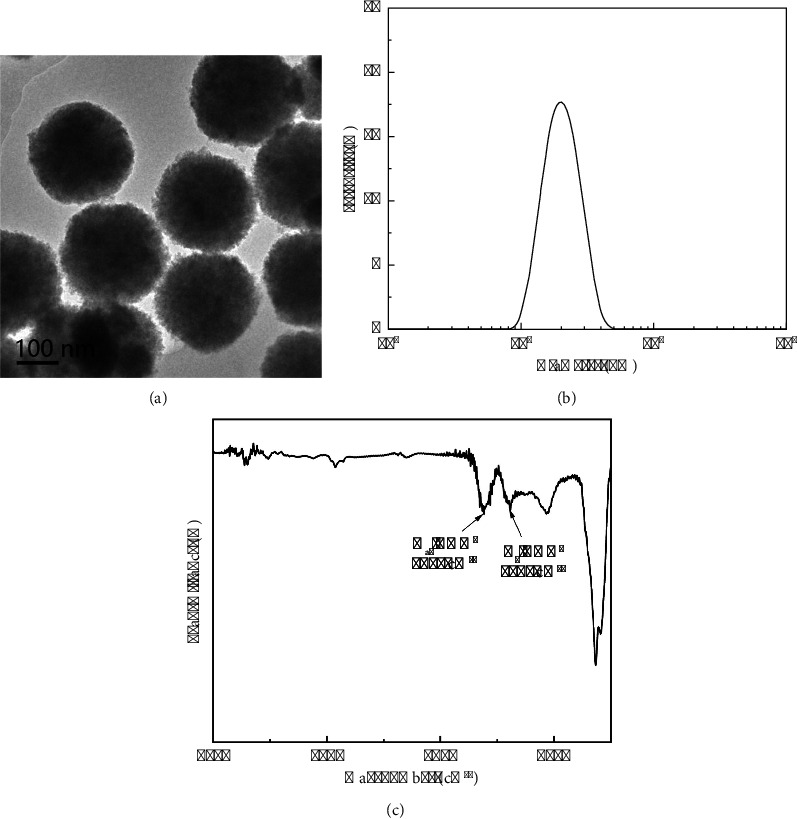
Characterization of MNPs. (a) TEM image demonstrates that the MNPs exhibit a consistent spherical shape. (b) NTA determined that the grain diameter of magnetic nanoparticles was 200 nm. (c) FTIR spectrum revealed the successful modification of two carboxyl groups on the MNPs.

A transmission electron microscope (TEM) was utilized to examine the morphology of MNPs. The TEM image demonstrates that the MNPs exhibit a consistent spherical shape, with an approximate diameter of 200 nm (Figure 2(a)). Nanoparticle tracking analysis (NTA) was utilized to determine the grain diameter of magnetic nanoparticles (MNPs). Our investigation revealed a singular peak exclusively at the magnitude of 200 nm. Nevertheless, the absence of peaks at alternative positions suggests that the synthesized nanomagnetic beads possess a consistent particle size, rendering them suitable for further investigations (Figure 2(b)). Moreover, the Fourier-transform infrared (FTIR) spectrum of unmodified magnetic nanoparticles (MNPs) is depicted in Figure 2(c). The FTIR peaks observed at 1617 cm^−1^ and 1385 cm^−1^ correspond to the asymmetric stretching mode and symmetric stretching mode of the COO- group, respectively. These findings indicate the successful modification of carboxyl groups on the MNPs during the synthesis process, enabling direct one-step protein modification on the MNPs without the need for additional functionalization in subsequent experiments.

### 3.2. Optimization of Incubation pH and Incubation Time

Initially, in order to apply the antibody onto the microplate, it is imperative to ascertain the suitable pH level of the PBS buffer. PBS buffer's pH range of gradients 4, 5, 6, 7, and 8 is considered. Subsequently, following the incubation period at 4°C, the residual solution within the microplate was eliminated, and the protein concentration was assessed utilizing the BCA kit (Thermo Fisher). In instances where the pH is suitable, the protein exhibits strong adherence to the microplate surface, resulting in a significantly reduced protein concentration. Conversely, when the pH is unsuitable, the protein connections are limited, leading to a substantial presence of antibodies in the liquid and consequently yielding a high measured protein concentration. It has been determined that the optimal pH value for this process is 7.

**Figure 3:**
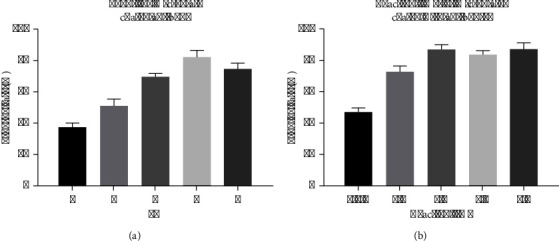
Optimization of the experimental conditions. (a) Optimization of incubation pH. (b) Optimization of incubation time.

Subsequently, the optimization of the reaction time for the microplate-linked antibody was conducted, with durations of 30 min, 1 h, 2 h, 3 h, and 4 h being tested. The antibody was diluted using the PBS solution that exhibited the optimal pH value determined earlier, followed by the measurement of protein concentration. The findings indicated that the most favorable incubation effect was attained at the 2 h mark, with negligible alterations observed beyond this duration. Consequently, 2 h was designated as the selected reaction time (Figure 3).

### 3.3. Optimization of Bifunctional Magnetic Beads

**Figure 4:**
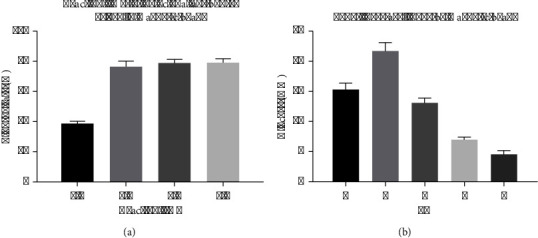
Optimization of bifunctional magnetic beads. (a) Reaction times of polyclonal antibodies linked to magnetic beads. (b) pH of invertase linked by magnetic beads.

The optimization of magnetic beads was conducted in two stages. First is the ligation of polyclonal antibodies using BB buffer. The optimization of the ligation pH for the polyclonal antibody was conducted. The pH level of BB utilized in the synthesis of invertase-MNPs-IgG conjugates (IMIcs) significantly impacts the coating efficiency. As evidenced by the data presented in Table 1, negative results were obtained from the agglutination test at pH 3 and pH 7, and positive results were obtained at pH 4 and pH 6, respectively. A strong positive result was obtained at pH 5 (Table 1). These findings suggest that the IgG in the antiserum was effectively coated onto the MNPs, and the conjugates exhibit a high capture efficiency for *P. aeruginosa*. Second is the optimization of the ligation duration of the polyclonal antibody. Generally, the efficacy of IMIc is significantly influenced by the quantity of IgG adhered to the MNPs. The duration of incubation greatly impacts the rate at which proteins bind during the synthesis of IMIc. The protein binding rate, as determined by the BCA kit, is depicted in Figure 4(a), where MNPs are saturated with antiserum and achieve a binding rate of 76.3% after 1 h. Consequently, 1 h was selected as the optimal incubation time condition. Lastly, the optimization of both the pH and time for the invertase ligation was performed. Due to the high cost and complexity associated with antibody preparation, we opted to employ unused magnetic beads, devoid of antibody attachment, to effectively optimize the pH and ligation duration for invertase ligation, thereby avoiding the wastage of previously antibody-bound magnetic beads.

Initially, invertase was immobilized onto magnetic beads at varying pH levels (3, 4, 5, 6, and 7), and the duration of immobilization was provisionally set at 1 h. Subsequently, the immobilized magnetic beads-invertase complex underwent two washes with PBS at pH 5. Following this, 20 microliters of the complex were introduced into a sucrose solution that had been previously prepared, and the resulting mixture was subjected to a reaction at 50°C for a duration of 10 minutes.

Subsequently, 10 microliters of the resulting liquid were analyzed using a glucose sensor. The labeling efficiency of invertase on magnetic beads is directly influenced by the pH of the MES buffer. The results of our study indicated that the glucose sensor's signal exhibited an increase as pH decreased, reaching its maximum at a pH of 4. Our analysis further revealed that invertase displayed a positive charge when below or near its isoelectric point, leading to its attraction towards the negatively charged groups present on the surface of magnetic beads. This interaction facilitated the formation of chemical bonds. Consequently, a pH of 4 was chosen as the optimal incubation condition for linking glycozymes. Based on previous research, it is generally recommended to set the connection time between invertase and magnetic beads at 1 hour to achieve optimal connection. However, in this study, the magnetic beads were preconnected with polyclonal antibodies. To enhance the spatial structure connection between invertase and magnetic beads, the connection time of invertase was extended to 2 hours during the preparation of the “polyclonal antigen-magnetic beads-invertase” complex. Subsequently, the polyclonal antibody-magnetic beads-invertase complex was blocked with BSA solution, fixed in PBS, and stored for future use (Figure 4(b)).

### 3.4. The Evaluation of Specificity

**Figure 5:**
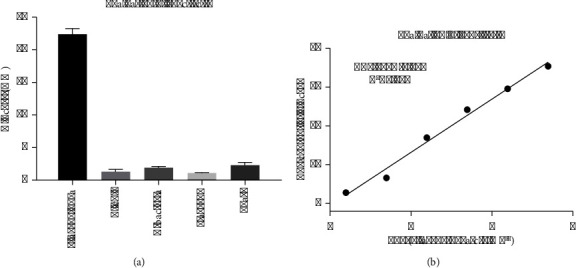
The evaluation of specificity and sensitivity. (a) The evaluation of specificity. (b) The evaluation of sensitivity.

The selectivity of PGM-POCT was assessed through the examination of 10^7^ CFU/mL of *P. aeruginosa* and other microorganisms, including *Escherichia coli*, *Klebsiellapneumoniae*, *Staphylococcus aureus*, and yeast. As depicted in Figure 5(a), solely *P. aeruginosa* exhibited a positive reaction, whereas the remaining 4 non-*P*. *aeruginosa* strains displayed low PGM signal. The consistent physical and chemical attributes of MNPs enhance the accuracy and precision of the detection analysis. Upon conjugation, the labeled samples exhibit favorable specificity. These characteristics guarantee the PGM-POCT system's reliable reproducibility and selectivity throughout the experimental process.

### 3.5. The Evaluation of Sensitivity

In order to validate the efficacy of the PGM-POCT assay in targeting *P. aeruginosa*, a range of *P. aeruginosa* standards with varying concentrations were examined under optimal conditions on human *P. aeruginosa* antibody-coated polystyrene 96-well microplates. IMIcs were utilized as the signal-generation tags, and the readings were obtained using a glucometer.  Figure 5(b) illustrates that the digital signals of the glucometer exhibit an increase corresponding to the rise in target *P. aeruginosa* levels in the sample. This observation aligns with the understanding that a higher concentration of *P. aeruginosa* results in the binding of a greater number of PGM-POCT through specific antigen-antibody interactions. A strong linear correlation between the PGM signal (mM) and glucose concentration (U mL^−1^) was observed within the dynamic working range of 2.46^∗^10^2^–2.46^∗^10^7^ CFU/ml. Furthermore, it was possible to accurately monitor concentrations as low as 6.8^∗^10 CFU/ml. The linear regression equation was Y (mM) = 3.417*x* − 2.076 (U·mL^−1^, *R*^2^ = 0.987).

### 3.6. Evaluation of the Actual Sample Test Effect

In this study, we applied a *P. aeruginosa* culture medium directly onto the bronchoscope, followed by rinsing the bronchoscope with 10 ml of normal saline. Subsequently, the obtained sample was subjected to detection using our method, resulting in a quantification of 7.8^∗^10^3^ CFU/ml. In addition, we performed the smearing of *P. aeruginosa*, followed by normal disinfection, wherein the disinfection time was halved and then reduced by 3/4. Using this device, the results obtained were as follows: no detectable glucose concentration, no detectable glucose concentration, and 4.1^∗^10^2^ CFU/ml, respectively.

## 4. Discussion

Various types of electricity-based glucose sensors are available, with the GM sensor being the most commonly utilized due to its numerous advantages including speed, sensitivity, accuracy, and portability. In addition, the combination of PGM-POCT with nanomaterials has been shown to enhance sensitivity and accuracy, making it valuable for clinical diagnosis. As demonstrated by Wang et al., the utilization of PGM in conjunction with a microfluidic chip enabled the simultaneous detection of the three types of hepatitis B virus nucleic acids, resulting in an enhanced detection sensitivity [14]. The detection limit reached as low as 10 pM. Furthermore, PGM-POCT requires minimal equipment and personnel, further highlighting its practicality and efficiency. Once the production of IMIc is consistently reliable, it presents a viable option for clinical application in resource-limited regions, particularly in the management of nosocomial infections, thereby narrowing the healthcare disparity between economically developed and underdeveloped areas.

In this study, microplates coated with capture antibodies were employed to concentrate *P. aeruginosa* in bronchoscopic specimens. The decision to utilize PGM-POCT for *Pseudomonas aeruginosa* was based on the practical clinical requirements. Within oncology hospitals, patients undergoing radiotherapy and chemotherapy are particularly vulnerable to infections and drug-resistant strains due to the immunosuppressive effects of these treatments. *P. aeruginosa* is a prevalent pathogen that frequently results in severe complications and mortality. In recent years, MNPs have gained popularity as nanomaterials in both basic research and clinical applications due to their advantageous characteristics, such as affordability, biocompatibility, and ease of use. MNPs have been modified by coating them with antibodies and invertase to create a bifunctional IMIc, which has streamlined detection processes and reduced detection times. In clinical practice, prompt evaluation of disinfection quality following bronchoscopy procedures can effectively minimize patient wait times, given the high volume of daily procedures. This is of particular significance in nations such as China, characterized by a high volume of hospital patients.

Currently, the detection of *P. aeruginosa* primarily relies on colony culture methods and next-generation sequencing (NGS) techniques. While colony culture is cost-effective and straightforward, its turnaround time exceeds 48 hours, which is often inadequate for the timely needs of bronchoscopy procedures. NGS is advancing rapidly in China and is recognized as a critical tool for pathogen detection. However, NGS has yet to receive approval from Chinese medical regulatory authorities (NMPA), and its cost remains above $500, imposing significant economic strain on patients. In contrast, PGM-POCT offers high sensitivity, ease of use, and objective results, with a single test costing less than $30. Nevertheless, PGM-POCT is still in the experimental development phase. Future research may focus on incorporating assembly line production and freeze-drying techniques for processing IMIc, which could substantially reduce both operational and storage costs. This approach represents a promising direction for subsequent research efforts.

## 5. Conclusion

This article presents a novel qualitative and quantitative immunoassay for point-of-care monitoring of *P. aeruginosa* utilizing PGM as a readout. In addition, biomolecules can be efficiently labeled and separated using MNPs in conjunction with a permanent magnet. The protein-modified MNPs demonstrate a strong selectivity and resistance to interference. Consequently, the newly developed PGM-ICA employing IMIc represents a promising and versatile approach for detecting *P. aeruginosa*, with potential applicability to other bacterial species through modification of the antibody.

## Figures and Tables

**Table 1:** The optimization of the ligation pH for the polyclonal antibody.

pH	Agglutination test
3	—
4	+
5	++
6	±
7	—

## Data Availability

The data used to support the findings of this study are available from the corresponding author upon reasonable request.
